# miR-96 Inhibits SV2C to Promote Depression-Like Behavior and Memory Disorders in Mice

**DOI:** 10.3389/fnbeh.2020.575345

**Published:** 2021-03-19

**Authors:** Lidong Sun, Donghao Bai, Maoguang Lin, Eerdenidalai, Li Zhang, Fengzhen Wang, Shangwu Jin

**Affiliations:** ^1^Outpatient Department, Ordos Fourth People’s Hospital, Ordos, China; ^2^Clinical Laboratory, Ordos Fourth People’s Hospital, Ordos, China

**Keywords:** microRNA-96, synaptic vesicle glycoprotein 2C, malondialdehyde, depression-like behaviors, memory disorders, superoxide dismutase

## Abstract

Accumulating evidence continues to emphasize the role of microRNAs as significant contributors to depression-like behavior and memory disorders. The current study aimed to investigate the mechanism by which miR-96 influences depression-like behavior and memory deficit in mice. A depression-like behavior and memory disorder mouse model was initially established by means of intraperitoneal injection with lipopolysaccharide. Memory deficits in the mice were evaluated using the Novel Object Recognition Test and Morris water maze experiments, whereas the Sucrose Preference Experiment and forced swimming experiments were performed to identify depression-like behavior in mice. The levels of tumor necrosis factor-α, malondialdehyde, superoxide dismutase, glutathione, and the monoamine transmitters 5-hydroxytryptamine and dopamine were subsequently detected in the serum. Reverse transcription-quantitative polymerase chain reaction and Western blot analysis evaluated the expression of miR-96 and SV2C expression in the CA1 hippocampal region of the mice. Finally, the relationship of miR-96 and SV2C was verified by dual-luciferase reporter gene assay. Our data indicated that the expression of miR-96 was increased, whereas that of SV2C was decreased in the CA1 region of mice exhibiting depression-like behavior and memory impairment. When miR-96 was downregulated or SV2C was overexpressed *via* intra-cerebroventricular injection with a miR-96 antagonist (miR-96 antagomir) or overexpression of SV2C vector, the Novel Object Recognition Test and sucrose preference index were increased, whereas the escape latency, the number of water maze platform crossings, and the immobility time of the mice were decreased. The serum levels of tumor necrosis factor-α, interleukin-1β, and malondialdehyde in the mouse CA1 region of mice were reduced, whereas the levels of superoxide dismutase and glutathione were elevated after the downregulation of miR-96 or overexpression of SV2C. Collectively, our study demonstrates that miR-96 negatively regulates the expression of SV2C, which consequently leads to depression-like behavior and memory impairment in mice. Our findings highlight the potential of miR-96-targeted therapeutics.

## Introduction

Depressive disorders have exhibited a progressive increase among younger age demographics and are often a detrimental contributor to serious impairments from both personal and social functioning points of view (Zhou et al., 2017). Early neonatal immune activation has been linked with depression-like behaviors in adult mice, whereas major depressive disorder has been reported to be a risk factor in memory impairment, illustrated by poor memory for positive reinforcing stimuli (Doosti et al., 2013; Sorenson et al., 2014). Memory impairment represents an early and disabling manifestation of multiple sclerosis, although the finer anatomical and biological mechanisms of this are still subjected to research investigation (Planche et al., 2017). Previous literature has argued that the prognosis of depression in conjunction with memory disorders remains poor due to the lack of effective therapeutic targets, highlighting the critical need to explore the mechanisms by which depression-like behaviors and memory disorders occur to identify new therapeutic targets.

The aberrant expression of microRNAs (miRNAs) has been implicated in depression, with certain miRNAs emerging as important regulators of synaptic function and memory (Wang et al., 2017, 2018). For example, the expression of miR-124 is involved in depression-like behavior (Gu et al., 2019; Lou et al., 2019). The downregulation of mR-34c has been shown to improve memory deficits by targeting vesicle-associated membrane protein 2 (Zhang et al., 2015). Previous literature has implicated miR-96 in the memory impairment observed in neonatal rats induced by sevoflurane. Higher concentrations of sevoflurane have been revealed to diminish the learning and memory performance of rats. Sevoflurane significantly upregulates the expression of miR-96, to the detriment of hippocampal neurons of neonatal rats. Furthermore, miR-96 may regulate insulin-like growth factor 1 receptor expression to influence the effects of sevoflurane on learning and memory impairment in rats (Xu et al., 2019). Previous studies have emphasized the abnormal expression of miR-96 as a factor involved in cognitive impairment and memory impairment in rats (Dwivedi, 2016; Liu et al., 2018). Despite the clear association between severe depressive disorders with memory impairment, no distinct research has been reported regarding the involvement of miR-96 in depression. Moreover, previous studies have suggested that the serotonin 5-hydroxytryptamine receptor 1B receptor in the brain is related to aggressive behavior, representing a crucial endophenotype associated with suicidal behavior and a notable element involved in regulating suicidal behavior in depression. Furthermore, studies have found an element (a-element) negatively regulated by miR-96 in the 5-hydroxytryptamine receptor 1B gene (Jensen et al., 2009). Hence, we asserted that miR-96 could be used as a potential biomarker of depressive behavior.

The synaptic vesicle glycoprotein 2 (SV2) family is comprised of three paralogs: SV2A, SV2B, and SV2C (Stout et al., 2019). SV2C is expressed in a variety of cell types, particularly dopaminergic, GABAergic, and cholinergic cells. The deletion of SV2C has been reported to trigger a decrease in striatal dopamine release, which may play a key role in the pathogenesis of depression (Rai et al., 2017). Moreover, increased dopamine transmission and running exercise can prevent anxiety- and depression-like behavior and memory disorders in rats (Lapmanee et al., 2017). Therefore, we speculate that SV2C is closely related to depression. Previous reports have suggested that a mutation in miR-96 may consequently lead to aberrant SV2 expression (Schluter et al., 2018); however, no in-depth research into the possible relationship between miR-96 and SV2C in depression has been conducted. Based on the exploration mentioned earlier of literate, we speculate that miR-96 may regulate depression-like behavior and memory disorder by targeting SV2C. To further elucidate the interaction between miR-96 and SV2C, changes in miR-96 and key protein molecules after lipopolysaccharide (LPS)-induced depression-like behavior and memory impairment were evaluated in mice. *In vivo* animal transfection technology was applied to assess miR-96 and SV2C expression in relation to depression-like behavior in mice, with the chief objective of identifying a novel therapeutic target for the treatment of depression-like behaviors and associated memory deficits.

## Materials and Methods

### Ethics Statement

All animal experiments were performed in accordance with the recommendations of the Guide for the Care and Use of Laboratory Animals of the National Institutes of Health. Additionally, the study protocol was approved by the Animal Care and Use Committee of Ordos Fourth People’s Hospital.

### Experimental Animal

One-hundred healthy specific pathogen-free male Institute of Cancer Research mice [aged 8–10 weeks old (Tang et al., 2019), weighing 33 ± 2 g] were purchased from Hunan SJA Laboratory Animal Co., Ltd. (Hunan, China). All mice were reared in a 12-h light cycle and provided with free access to food and drink under temperature-controlled conditions at 22 ± 2°C and 60 ± 5% relative humidity.

### Experiment Grouping and Processing

Ten mice were initially assigned to the sham group and were injected intraperitoneally with saline. The remaining mice were administered with an intraperitoneal injection with LPS (L8880-10 mg; Beijing Solarbio Technology Co., Ltd., Beijing, China) at a dose of 0.5 mg/kg to induce depression-like behaviors and memory deficits (Li et al., 2017). All mice were housed in isolation. Next, 10 modeled mice were selected as the control group, with the remaining model mice injected with the corresponding treatments into the lateral ventricle: negative control (NC) agomir (NC of miR-96 overexpression), miR-96 agomir (miR-96 overexpression), NC antagomir (NC of miR-96 antagomir), miR-96 antagomir, oe-NC (NC of SV2C overexpression) (Table 1), and/or oe-SV2C (SV2C overexpression). All drugs mentioned earlier and plasmids were purchased from Genomeditech (Shanghai, China). The drugs mentioned earlier were dissolved in artificial cerebrospinal fluid, with the mice anesthetized using pentobarbital (5 mg/kg) and placed in a stereotactic frame with a flat skull position. A stainless-steel guide cannula (Plastics One, Roanoke, VA, United States) was implanted into the bilateral ventricles based on the stereotactic coordinates of the mouse brain as follows: anterior–posterior location of the lateral ventricle −1.06 mm; lateral–medial + 1.75 mm; and dorsal–ventral 2.00 mm. Each catheter was wrapped with a dummy catheter to maintain sterility. The guide cannula was fixed to the skull with dental cement, and a stainless-steel tube was inserted to maintain the patency for the microinjection. The treatments mentioned earlier were microinjected into the dorsal ventricle, with the injection cannula withdrawn in a slow and progressive manner after being held in place for 5 min to avoid reflux. The subsequent testing experiments were performed 7 days post-injection.

**TABLE 1:** RT-qPCR primer sequences.

Gene	Primer sequences
miR-96	Forward: 5′-GCCCGCTTTGGCACTAGCACATT-3′
	Reverse:5′-GTGCAGGGTCCGAGGT-3′
SV2C	Forward: 5′-CAGAGAGGAGGGCTGATGAG-3′
	Reverse:5′-CTGCACTGGGTAGCACGAA-3′
U6	Forward: 5′-TGCGGGTGCTCGCTTCGGCAGC-3′
	Reverse:5′-CCAGTGCAGGGTCCGAGGT-3′
β-actin	Forward: 5′-CAGAGCCTCGCCTTTGCC-3′
	Reverse:5′-GTCGCCCACATAGGAATC-3′

### Memory Impairment Behavior Test in Mice

Novel Object Recognition Test (NORT) was performed as follows: The experiment was performed in a soundproof plexiglass box measuring 25 × 25 × 25 cm. The behavior of mice was recorded using a built-in video camera. The experiment consisted of an adaptation period, a learning training period, and a testing period. During the adaptation period, each mouse was initially conditioned in an empty box for 10 min and subsequently returned to its home cage. During the learning and training period, two objects with the same shape were placed in two of the three positions on the diagonal base of the box. Each mouse was returned to the box and allowed 10 min of free exploration. Video recording was used to calculate the percentage of the mouse’s exploration time in proportion to one object relative to the total exploration time, i.e., novel position preference. During the testing period, one of the objects in the box was replaced with a novel object, which had a different color and shape from the original object. After 1 h in the home case, the mice were returned to the box to explore both old and new objects for 5 min. Behavior at this stage was used to calculate the percentage of the mouse’s exploration time for new objects relative to the total exploration time, which was used to evaluate the working memory of the mouse.

Morris water maze experiment: A mouse water maze real-time monitoring system was used to monitor the activity of the mice. The experiment was divided into a positioning and navigation phase and a space exploration phase. During the positioning and navigation phase, mice were trained three times a day for a total of 6 days, with each training session lasting 90 s detecting time plus 15 s adapting time before and after each training. In the event that the mouse discovered the platform within the time range, this was considered as a successful platform search with its escape latency recorded. In the event the mouse failed to locate the platform, its escape latency was recorded as 90 s. In the space exploration stage, the platform was removed, with the animals placed into the water facing the pool wall in the diagonal quadrant of the original platform quadrant. The total run length, average speed, and the speed and the frequency of crossing the original platform location were recorded during 90 s.

### Depression-Like Behavior Test in Mice

Sucrose Preference Experiment (SPT): Before the SPT, two identical water bottles were placed in each cage, and the mice were trained to drink from the two water bottles. During the first 24 h, both bottles contained the same amount (100 ml) of 1% sucrose solution, and in the second 24 h, one bottle contained (100 ml) 1% sucrose solution, and the other bottle contained (100 ml) water. The mice were placed on a fasting diet for food and water for a period of 20 h after adaptive training. On the next day, the experiment began. One bottle of 100 ml of sucrose water and one 100-ml bottle of water were randomly placed in each cage, removed after 1 h, and reweighed to record the volumes of fluid consumed. Sugar preference value = sugar consumption/(sugar consumption + water consumption) × 100%.

Forced swimming experiment: After the sugar and water preference experiment, mice were placed in a cylindrical plexiglass swimming tank with a height of 20 cm, a diameter of 12 cm, and a water depth of 10 cm. The water temperature was (24 + 1)°C. Behavior was recorded for 6 min; the accumulated immobility time within the final 4 min was calculated. The “immobility” criterion was met when the mouse stopped struggling in the water, floated, or had only small limb movements to keep their heads floating on the water. The rest time was recorded and monitored manually by the experimenter (Chen et al., 2020).

### Enzyme-Linked Immunosorbent Assay

The mice were anesthetized after the administration of a lethal dose of pentobarbital (50 mg/kg). After decapitation, the hippocampal tissues were collected promptly, after which the brain tissues of the CA1 region were quickly separated from the ice box and subsequently frozen in liquid nitrogen and stored at −80°C. The mouse brain tissues in the CA1 region from each group were collected to prepare tissue homogenate. The enzyme-linked immunosorbent assay (ELISA) has strictly followed the steps of the kit instructions to draw a standard curve and measure the levels of inflammatory factors interleukin (IL)-1β (69-86579), tumor necrosis factor-α (TNF-α) (69-22452), and oxidative stress factors malondialdehyde (MDA) (69-40588), superoxide dismutase (SOD) (69-35263), glutathione (GSH) (69-57683), 5-hydroxyindole acetic acid (5-HT) (69-40551), and dopamine (DA) (69-56813) in the brains of mice in each group. The kits mentioned earlier were purchased from Wuhan Mskbio Biological Technology (Wuhan, China) (Zhao et al., 2010).

### Reverse Transcription-Quantitative Polymerase Chain Reaction

The total RNA was extracted from brain tissues in the CA1 region based on the instructions of the TRIzol kit (Invitrogen, Carlsbad, CA, United States). The brain tissue extraction method was performed identically to that of ELISA. The primers were designed and synthesized by Aoke Biotechnology Co., Ltd. (Wuhan, Hubei, China) (Table 1). The reaction solution was then subjected to real-time PCR in accordance with the instructions of the SYBR^®^ Premix Ex Taq^TM^ II Kit (RR820A, Qicha Biological Technology Co., Ltd., China). The stem-loop structure primers were used for reverse transcription of miR-96 mature RNA. The quantitative PCR (qPCR) experiments were performed using an ABI 7500 real-time (RT)-qPCR machine (Prism^®^ 7300, Shanghai kunke Instrument Equipment Co., Ltd., Shanghai, China). The U6 and β-actin were regarded as internal references for miR-96 and SV2C. The 2^–ΔΔCt^ was applied to calculate the difference between the experimental group and the control group, as follows: ΔΔCT = ΔCt experimental group – ΔCt control group, where ΔCt = Ct target gene – Ct internal reference gene.

### Western Blot Analysis

Total protein from the mouse brain CA1 region was extracted in strict accordance with the instructions of the radio-immunoprecipitation assay lysate kit (R0010, Beijing Solarbio Science & Technology Co., Ltd., Beijing, China). Protein concentration was determined using a bicinchoninic acid kit (20201ES76, Beijing Solarbio Science & Technology Co., Ltd., Beijing, China). Quantification was conducted based on different concentrations. After protein separation *via* polyacrylamide gel electrophoresis, the protein was transferred to a polyvinylidene fluoride membrane using a wet transfer method, followed by blockade with 5% bovine serum albumin for 1 h at room temperature. After that, the membrane was incubated at 4°C overnight with the diluted primary antibodies SV2C (ab33892, Abcam Inc., Cambridge, United Kingdom), glyceraldehyde-3-phosphate dehydrogenase (ab181602, Abcam Inc.). After three Tris-buffered saline with Tween washes (5 min per wash), the membrane was incubated with horseradish peroxidase-labeled goat anti-rabbit immunoglobulin G (ab150077, Abcam Inc.). Enhanced chemiluminescence (WBKLS0100, Millipore, Billerica, MA, United States) was used for development. ImageJ 1.48 μ software (National Institutes of Health) was used for quantitative protein analysis, quantitative protein analysis = gray value of each protein/gray value of internal reference β-actin.

### Bioinformatics Methods

The downstream target genes of miR-96 were predicted through TargetScan (cumulative weighted context + + score ≤ 0.25)^1^, miRDB (target score > 85)^2^, RAID^3^, starBase (pancancerNum > 5)^4^, and DIANA TOOLS (miTG score > 0.9)^5^ databases. The depression-related gene expression dataset GSE84185 was obtained through the Gene Expression Omnibus database^6^, which comprised 96 samples from blood, dentate gyrus, and anterior cingulate cortex. Among these, the 48 samples unrelated to our study were removed, leaving eight normal samples and eight depression samples for each tissue. In light of our study objective and emphasis on brain tissues, blood sample data were excluded. The differential analysis between the normal and diseased cortex of dentate gyrus and anterior cingulate gyrus was performed using R language “limma” package^7^ with the threshold value set at |logFC| > 0.3 and *p* < 0.05. The results indicated that the sequencing quality of the anterior cingulate cortex was higher than that of the dentate gyrus. The intersection of miR-96 downstream target genes and differentially expressed genes in the anterior cingulate cortex of expression dataset GSE84185 was used to construct a Venn map to identify the key genes in connection with the key findings from existing literature.

### Dual-Luciferase Reporter Gene Assay

The biological website^8^ and dual-luciferase reporter gene assay were used to predict and verify the relationship between miR-96 and SV2C. The wild-type (WT) promoter of SV2C was constructed in a dual-luciferase reporter gene vector (PGLO-SV2C WT) with the mutants (MUT) promoter with mutation sites binding to miR-96 (PGLO-SV2C MUT) constructed in vector. The reporter plasmids were co-transfected with miR-96, SV2C overexpression plasmids, and negative control plasmids into the HEK293T cells. The cells were lysed 24 h after transfection, centrifuged at 12,000 rpm/min for 1 min, followed by a collection of the supernatant. Luciferase activity was detected using the dual-luciferase reporter assay system (E1910, Promega, Madison, WI, United States). Each cell sample was added with a 100-μl working solution of firefly luciferase to detect firefly fluorescence signal, followed by the addition of a 100-μl renilla luciferase working solution to detect renilla fluorescence signal. Firefly fluorescence signal and renilla fluorescence signal were used to obtain relative luciferase activities.

### Statistical Analysis

All statistical analyses were performed using GraphPad Prism (GraphPad Software, La Jolla, CA, United States). Measurement data were expressed as the mean ± standard deviation. Data between two groups conforming to normal distribution and homogeneity of variance were compared using an unpaired *t*-test; otherwise, a Welch’s *t*-test was used. Statistical analysis in relation to time-based measurements within each group was performed using repeated-measures analysis of variance (ANOVA), followed by Bonferroni’s *post hoc* test. Two-way ANOVA was applied for dual-luciferase reporter gene assay, followed by Bonferroni’s *post hoc* test. To ascertain the effect of miR-96 on SV2C and depression-like behaviors and memory disorders in mice, the expression of SV2C, behavioral data, NORT index, and the content of key proteins after SV2C overexpression were analyzed by one-way ANOVA, followed by the application of a Tukey’s *post hoc* test. A value of *p* < 0.05 was considered to be indicative of a statistically significant difference. The study design schematics are depicted in Supplementary Figure 1.

## Results

### miR-96 Was Upregulated in the Brain CA1 Region of Lipopolysaccharide-Induced Mice

**FIGURE 1:**
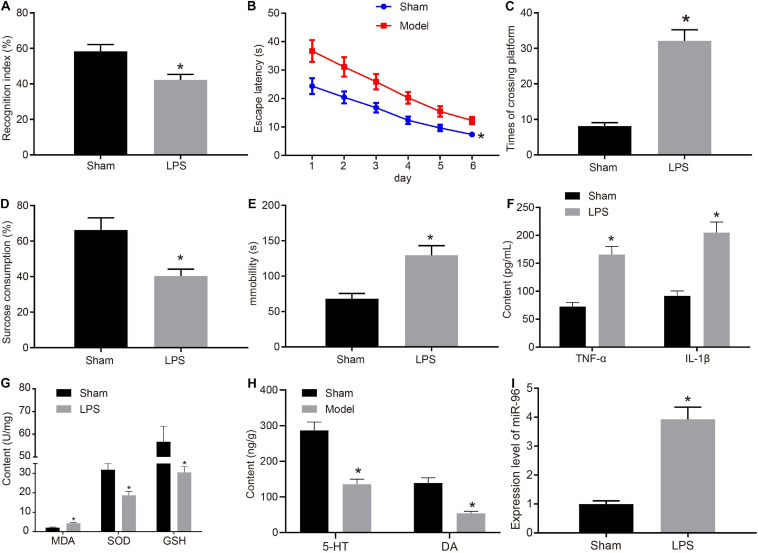
miR-96 was highly expressed in CA1 region of brain in mice with depression-like behavior and memory impairment. **(A)** NORT index of mice after LPS induction, *N* = 10, *t* (18) = 10.38. **(B)** Escape latency of mice after LPS induction in Morris water maze experiment, *N* = 10, *F*(5,108) = 7.897. **(C)** Frequency of crossing platform of mice after LPS induction in Morris water maze experiment, *N* = 10, *t* (18) = 15.14. **(D)** Statistical results of sucrose preference experiment of mice after LPS induction, *N* = 10, *t* (18) = 10.50. **(E)** Immobility time of mice after LPS induction in forced swimming experiment, *N* = 10, *t* (18) = 12.56. **(F)** Levels of inflammatory factors TNF-α and IL-1β in CA1 region of brain of mice after LPS induction measured by ELISA [*N* = 10, t_TNF–α_ (18) = 18.34, t_IL–1β_ (12.86) = 17.20]. **(G)** Levels of MDA, SOD, and GSH in brain of mice after LPS induction, [*N* = 10, t_MDA_ (18) = 13.04, t_SOD_ (18) = 11.24, t_GSH_ (12.27) = 11.18]. **(H)** Levels of 5-HY and DA in brain CA1 region after LPS induction [*N* = 10, t_5–HY_ (18) = 17.27, t_DA_ (11.91) = 17.25]. **(I)** RT-qPCR detection of miR-96 expression in mouse CA1 region after LPS induction, *N* = 10, *t* (10.04) = 21.29. *p < 0.05 vs. sham-operated mice. Measurement data were expressed as mean ± standard deviation. If data were in compliance with normal distribution and homogeneity of variance, unmatched data between two groups were analyzed using an unpaired *t*-test. Statistical analysis in relation to time-based measurements within each group was realized using repeated-measures ANOVA, followed by Bonferroni’s *post hoc* test. Cell experiment was independently repeated three times.

Depression-like behaviors and memory deficits in mice were induced using LPS treatment. To detect depression-like behavior and memory deficit in the mice, we assessed memory and learning using NORT (Figure 1A) and Morris water maze experiments (Figures 1B,C). Sucrose preference experiment (Figure 1D) and forced swimming experiment (Figure 1E) were performed to identify depression-like behavior in mice, respectively, as anhedonia and behavioral despair. The results obtained indicated that compared with the sham-operated mice, the LPS-treated mice had reduced NORT index and sucrose preference index while increased escape latency, platform crossing, and immobility time. ELISA (Figure 1F) results indicated that compared with the sham-operated mice, the TNF-α and IL-1β levels in the brain CA1 region were elevated in LPS-treated mice.

At the same time, molecular markers were used to determine oxidative stress. In contrast to sham-operated mice, the MDA level in the brain was enhanced, and the SOD and GSH levels were reduced in LPS-treated mice (Figure 1G). ELISA was conducted to detect the monoamine transmitters 5-HT and DA in the mouse brain (Figure 1H). The results obtained indicated that both the 5-HT and DA levels were decreased in the CA1 hippocampus region of LPS-treated mice compared with sham-operated mice. Existing literature has highlighted the relationship between miR-96 and circadian rhythm regulation (Xu et al., 2007), whereas the disruption of circadian rhythm has long been implicated in the pathophysiology of major depression (Saus et al., 2010). Hence, we speculated that miR-96 was related to depression-like behavior and memory impairment in mice. To determine the expression of miR-96, RT-qPCR was performed to determine the expression of miR-96 in the CA1 region of mice. Results (Figure 1I) revealed that the level of miR-96 was higher in LPS-treated mice than that in sham-operated mice. The findings mentioned earlier demonstrated that LPS induced depression-like behavior and memory disorders in mice, and miR-96 was upregulated in the CA1 region of the brain in LPS-treated mice.

### Downregulated miR-96 Alleviated Depression-Like Behavior and Memory Impairment in Lipopolysaccharide-Treated Mice

**FIGURE 2:**
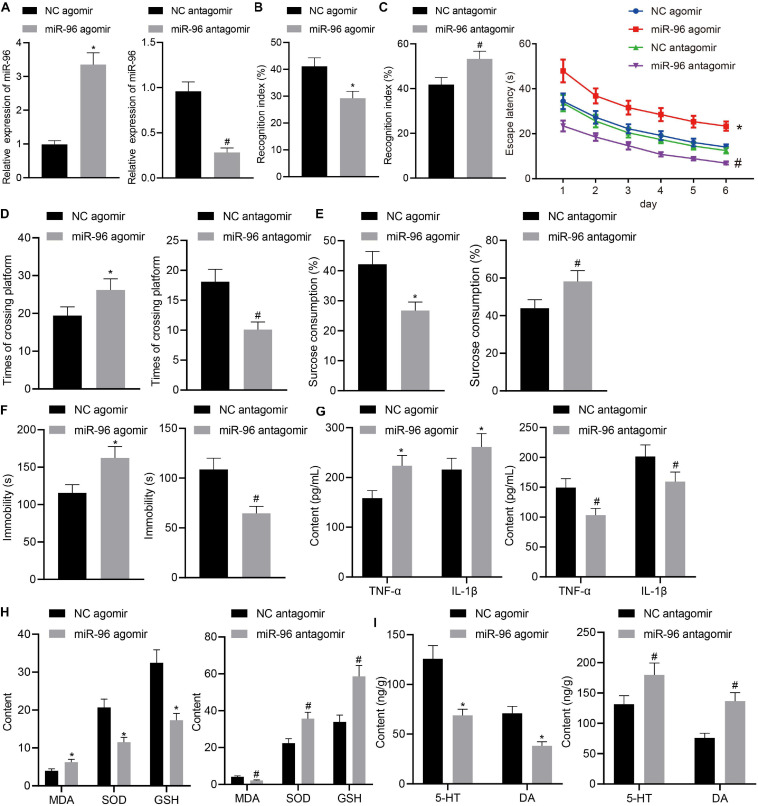
Downregulation of miR-96 relieved depression-like behavior and memory impairment in LPS-treated mice. LPS-treated mice were injected with NC agomir, miR-96 agomir, NC antagomir, or miR-96 antagomir. **(A)** RT-qPCR detection of miR-96 expression in mouse CA1 region [*N* = 10, t_agomir_ (10.89) = 20.41, t_antagomir_ (12.84) = 18.69]. **(B)** NORT index of mice [*N* = 10, t_agomir_ (18) = 9.352, t_antagomir_ (18) = 7.684]. **(C)** Escape latency time of mice in Morris water maze experiment [*N* = 10, *F*(15,216) = 2.715]. **(D)** Frequency of crossing platform by Morris water maze experiment [*N* = 10, t_agomir_ (18) = 5.747, t_antagomir_ (18) = 10.35]. **(E)** Sucrose preference index of mice [*N* = 10, t_agomir_ (18) = 9.588, t_antagomir_ (18) = 6.163]. **(F)** Swimming immobility time of mice in forced swimming experiment [*N* = 10, t_agomir_ (18) = 7.893, t_antagomir_ (18) = 10.47]. **(G)** Levels of inflammatory factors TNF-α and IL-1β in CA1 region of brain in mice detected by ELISA [*N* = 10, t_agomir, TNF–α_ (18) = 7.996, t_agomir, IL–1β_ (18) = 4.068, t_antagomir, TNF–α_ (18) = 7.685, t_antagomir, IL–1β_ (18) = 5.275]. **(H)** Levels of MDA, SOD, and GSH in CA1 region of brain in mice [*N* = 10, t_agomir, MDA_ (18) = 7.679, t_agomir, SOD_ (18) = 11.54, t_agomir, GSH_ (18) = 12.40, t_antagomir, MDA_ (18) = 10.83, t_antagomir, SOD_ (18) = 10.07, t_antagomir, GSH_ (18) = 11.21]. **(I)** Levels of 5-HT and DA in hippocampal CA1 region of mice [*N* = 10, t_agomir, 5–HY_ (18) = 12.21, t_agomir, DA_ (18) = 12.82, t_antagomir, 5–HY_ (18) = 6.327, t_antagomir, DA_ (18) = 11.87]. *p < 0.05 vs. LPS-treated mice injected with NC agomir, #*p* < 0.05 vs. LPS-treated mice injected with NC antagomir. Measurement data were expressed as mean ± standard deviation. When data were in compliance with normal distribution and homogeneity of variance, unmatched data between two groups were compared using unpaired *t*-test. Statistical analysis in relation to time-based measurements within each group was realized using repeated-measures ANOVA, followed by Bonferroni’s *post hoc* test. Cell experiment was independently repeated three times.

To ascertain the effects of miR-96 on depressive behavior and memory impairment in mice, LPS-treated mice were injected with NC agomir, miR-96 agomir, NC antagomir, and miR-96 antagomir. The RT-qPCR (Figure 2A) result demonstrated that miR-96 expression was increased in LPS-treated mice injected with miR-96 agomir and was reduced in LPS-treated mice injected with miR-96 antagomir. Next, we performed NORT (Figure 2B), Morris water maze (Figures 2C,D), sucrose preference (Figure 2E), and forced swimming experiments (Figure 2F). The results revealed that the NORT index and sucrose preference index were reduced, whereas the escape latency, the times of crossing the platform, and immobility time were increased in LPS-treated mice injected with miR-96 agomir, which was opposite to findings in LPS-treated mice injected with miR-96 antagomir. As depicted in Figures 2G,H, the levels of TNF-α, IL-1β, and MDA were enhanced, and SOD and GSH levels were reduced in LPS-treated mice by injection with miR-96 agomir, whereas a contrasting trend was identified in the LPS-treated mice injected with miR-96 antagomir. The ELISA results (Figure 2I) revealed that the injection of miR-96 agomir decreased the level of 5-HT and DA hippocampus of LPS-treated mice, whereas injection with miR-96 antagomir increased their concentrations. Based on our findings, miR-96 downregulation could attenuate depression-like behavior and memory impairment in LPS-treated mice.

### miR-96 Targeted SV2C

**FIGURE 3:**
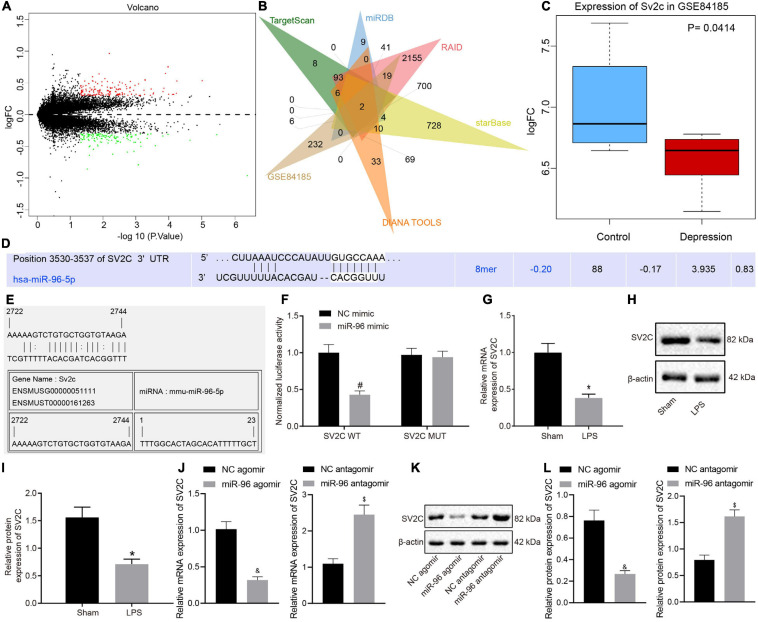
SV2C is targeted by miR-96. **(A)** Volcano map for the anterior cingulate cortex of gene expression dataset GSE84185, where red dots indicate significantly upregulated genes, green dots indicate significantly downregulated genes, and black dots indicate genes with no significant difference; **(B)** Venn map of miR-96 downstream target genes predicted by TargetScan, miRDB, RAID, starBase, and DIANA TOOLS and differentially expressed genes in anterior cingulate cortex of expression dataset GSE84185, the intersection genes are WDR82 and SV2C. **(C)** Expression of SV2C in normal tissues and depression model in GSE84185. **(D)** TargetScan prediction of the binding sites between miR-96 and SV2C. **(E)** RNA22 website predicts binding site of miR-96 with SV2C. **(F)** Validation targeting relationship between miR-96 and SV2C by dual-luciferase report gene assay [*N* = 3, *F*(1,8) = 30.06]. **(G)** RT-qPCR detection of SV2C mRNA expression in mice after LPS induction [*N* = 10, *t* (11.76) = 14.56]. **(H,I)** Western blot analysis of SV2C protein expression in mice after LPS induction [*N* = 10, *t* (12.9) = 13]. **(J)** RT-qPCR detection of SV2C mRNA expression in LPS-treated mice after miR-96 alteration [*N* = 10, t_agomir_ (12.31) = 19.87, t_antagomir_ (18) = 14.46]. **(K,L)**, Western blot analysis of SV2C protein expression in LPS-treated mice after miR-96 alteration [*N* = 10, t_agomir_ (10.99) = 15.57, t_antagomir_ (18) = 16.78]. *p < 0.05 vs. sham-operated mice, #*p* < 0.05 vs. NC mimic, &*p* < 0.05 vs. LPS-treated mice injected with NC agomir, $*p* <0.05 vs. LPS-treated mice injected with NC antagomir. Measurement data were expressed as mean ± standard deviation. When data were in compliance with normal distribution and homogeneity of variance, unmatched data between two groups were compared using an unpaired *t*-test. Comparisons among multiple groups in **(E)** were conducted by two-way ANOVA, followed by Bonferroni’s *post hoc* test. Cell experiment was independently repeated three times.

Research has indicated that miRNA mainly regulates downstream target genes in a post-transcriptional manner, ultimately controlling numerous biological processes. Through scrutiny of the databases of TargetScan, miRDB, RAID, starBase, and DIANA TOOLS, 445, 326, 3,585, 1,842, and 444 target genes of miR-96 were obtained, respectively. Differential analysis of depression-related gene expression dataset GSE84185 revealed 3,400 and 280 genes from dentate gyrus and anterior cingulate cortex, and the number of differentially expressed genes in dentate gyrus greatly exceeded that in the anterior cingulate cortex, which may potentially be due to the relatively poor sequencing quality of dentate gyrus tissues (Figure 3A). The intersection of miR-96 downstream target genes and differentially expressed genes in the anterior cingulate cortex of expression dataset GSE84185 showed two key genes, namely WDR82 and SV2C (Figure 3B). Previous studies have shown that SV2C regulates dopamine release and tissue dopamine content, and the genetic deletion of SV2C leads to dopamine depletion (Dardou et al., 2013; Dunn et al., 2017), which consequently can result in anxiety and depression-like behavior and memory deficits in rats (Lapmanee et al., 2017). We extracted the expression data of SV2C from the expression dataset GSE84185, the result of which revealed that the expression of SV2C was significantly diminished in the setting of depression (Figure 3C). TargetScan prediction revealed the existence of a binding site between miR-96 and SV2C (Figure 3D). Through the RNA22 website^9^, we found that there were also binding sites between miR-96 and SV2C (Figure 3E). The dual-luciferase reporter gene assay results showed that the luciferase activity in SV2C WT was inhibited by miR-96 mimic, whereas that of SV2C MUT was not affected (Figure 3F). RT-qPCR and Western blot analysis results indicated that the expression of SV2C mRNA and protein was reduced in LPS-treated mice (Figures 3G–I). After treatment with miR-96 antagomir, the expression of SV2C was increased in LPS-treated mice, whereas the miR-96 agomir downregulated SV2C expression (Figures 3J–L). Altogether, the results demonstrated that miR-96 could target and negatively regulate the expression of SV2C.

### miR-96 Downregulation Relieved Depression-Like Behavior and Memory Impairment in Mice by Upregulating SV2C

**FIGURE 4:**
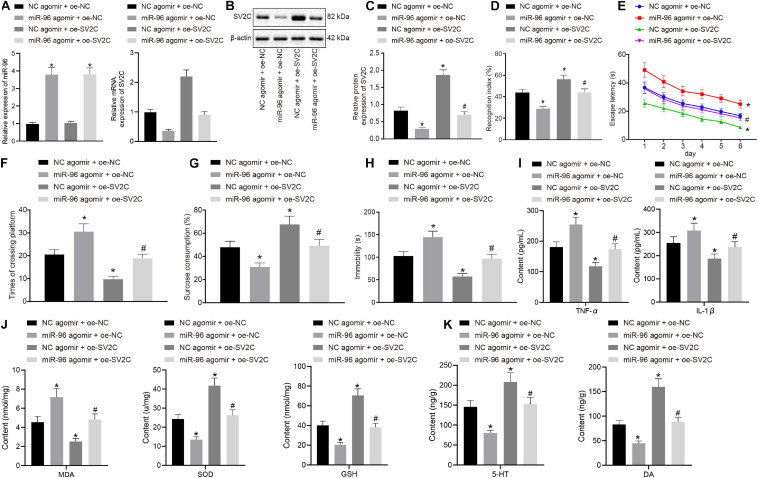
miR-96-targeted SV2C overexpression relieved depression-like behavior and memory impairment in mice. LPS-treated model-operated mice were injected with NC agomir + oe-NC, miR-96 agomir + oe-NC, NC agomir + oe-SV2C, or miR-96 agomir + oe-SV2C. **(A)** RT-qPCR detection of miR-96 expression and SV2C mRNA expression in brain CA1 region of mice [*N* = 10, F_MiR–96_ (3,36) = 333.8, F_SV2C_ (3,36) = 321.3]. **(B,C)** Western blot analysis of SV2C protein expression in brain CA1 region of mice [*N* = 10, *F*(3,36) = 420]. **(D)** NORT index of mice [*N* = 10, *F*(3,36) = 144.2]. **(E)** Escape latency time of mice in Morris water maze experiment [*N* = 10, *F*(15,216) = 1.899]. **(F)** Frequency of crossing platform in Morris water maze experiment [*N* = 10, *F*(3,36) = 135.7]. **(G)** Sucrose preference index of mice [*N* = 10, *F*(3,36) = 74.91]. **(H)** Swimming immobility time of mice by forced swimming experiment [*N* = 10, *F*(3,36) = 122.9]. **(I)** Levels of inflammatory factors TNF-α and IL-1β in CA1 region of brain in mice detected by ELISA [*N* = 10, F_TNF–α_ (3,36) = 96.16, F_IL–1β_ (3,36) = 37.39]. **(J)** Levels of MDA, SOD, and GSH in CA1 region of brain in mice [*N* = 10, F_MDA_ (3,36) = 88.85, F_SOD_ (3,36) = 165.2, F_GSH_ (3,36) = 200.7]. **(K)** Level of 5-HY and DA in brain CA1 region of mice [*N* = 10, F_5–HY_ (3,36) = 88.85, F_DA_ (3,36) = 206.1]. *p < 0.05 vs. LPS-treated mice injected with NC agomir + oe-NC, #*p* < 0.05 vs. LPS-treated mice injected with miR-96 agomir + oe-NC. Measurement data were expressed as mean ± standard deviation. Comparisons among multiple groups were conducted by ANOVA, followed by a Tukey’s *post hoc* test. Statistical analysis in relation to time-based measurements within each group was realized using repeated-measures ANOVA, followed by Bonferroni’s *post hoc* test. Cell experiment was independently repeated three times.

LPS-treated depression model mice were injected with NC agomir + oe-NC, miR-96 agomir + oe-NC, NC agomir + oe-SV2C, or miR-96 agomir + oe-SV2C to investigate the regulation of miR-96 on SV2C as well as its effect on depression-like behavior and memory impairment in mice. As depicted in Figures 4A–C, SV2C mRNA and protein expression in the CA1 region of LPS-treated mice was reduced by miR-96 agomir, which was negated by additional treatment of oe-SV2C, whereas it was increased in LPS-treated mice by subsequent oe-SV2C administration. As illustrated in Figures 4D–H, the NORT index and sucrose preference index scores were reduced, whereas the escape latency, platform crossing, and immobility times were increased in LPS-treated mice by miR-96 agomir, whereas opposite effects were seen in LPS-treated mice after overexpression of SV2C. Moreover, the effects of miR-96 agomir were reversed by oe-SV2C.

Enzyme-linked immunosorbent assay (Figure 4I) displayed that miR-96 agomir elevated the levels of TNF-α and IL-1β in the CA1 hippocampus region of LPS-treated mice, whereas an opposite trend was observed after treatment with overexpressed SV2C. Furthermore, the effects of miR-96 agomir on the levels of TNF-α and IL-1β were abrogated by oe-SV2C. Meanwhile, the MDA level was enhanced, and SOD, GSH, 5-HT, and DA levels were reduced in LPS-treated mice with miR-96 agomir treatment, with an opposite observation made after the overexpression of SV2C. The treatment with oe-SV2C reversed the effects of miR-96 agomir on the levels of MDA, SOD, GSH, 5-HT, and DA (Figures 4J,K). Thus, based on our results, inhibition of miR-96 alleviated depressive-like behaviors and memory disturbance in mice *via* SV2C.

## Discussion

Depression represents a global psychiatric illness afflicting millions (Su et al., 2019). The depression-like behavior phenotype was an area of particular research interest in connection with the pathogenesis of depression and antidepressant therapy (Huang et al., 2020). Numerous memory disorders brought on by neurodegenerative diseases, traumatic brain injury, vascular disease, and abnormal brain development share common features of memory-related pathology (Sun et al., 2015). In addition, recent research has emphasized that miRNAs are related to memory impairment in humans (Saab and Mansuy, 2014). Hence, we set out to elucidate the roles and related mechanism of miR-96 in depression. Key observations made during the current study indicated that miR-96 could promote the development of memory disorders and depression-like behaviors and memory deficits by targeting SV2C, thus aggravating depression-like symptoms.

Our initial findings demonstrated that miR-96 was expressed at a high level, whereas the expression of SV2C was lowly expressed in the hippocampus of mice with depression-like behaviors and memory deficits. A previous report demonstrated that miR-96 and miR-182 were highly expressed and involved in circadian rhythm regulation (Xu et al., 2007). Furthermore, disruption of circadian rhythms has long been implicated in the pathophysiology of major depression (Saus et al., 2010). SV2C represents an N-glycosylated protein that is concentrated on small synaptic vesicles and is also found on microvesicles in adrenal chromaffin cells (Janz and Sudhof, 1999). Existing literature has illustrated the role of SV2C as a member of the synaptic vesicle 2 protein family that exhibits a particular pattern of brain expression, with enriched expression in several basal ganglia nuclei (Dardou et al., 2013). SV2C deletion resulted in dopamine loss and sports injury, which triggered anxiety- and depression-like behavior and memory disorders in rats (Lapmanee et al., 2017; Rai et al., 2017). The evaluation of cognitive function is based mainly on locomotor activity, spatial memory, and anxiety (Manukyan et al., 2020); the Morris water maze and NORT tests revealed that locomotor and behavioral activity was impaired with lower miR-96 levels. Therefore, in concert with the previously published reports, we put forth out findings that demonstrate that the expression of miR-96 and SV2C is altered in mice induced with depression-like behaviors and memory disorders, highlighting their roles in depression. To further uncover the deeper mechanisms of miR-96 and SV2C in depression, in the subsequent experiments, we used a series of bioinformatics methods and dual-luciferase reporter gene assay, results of which showed that miR-96 targeted SV2C in mice.

SV2C is one of the three homologous proteins in the SV2 family. Various previous reports have indicated that SV2A regulates the release of neurotransmitters through a variety of mechanisms. At present, prevailing hypotheses have strongly linked the pathogenesis of depression with biogenic monoamine neurotransmitters. Certain studies have indicated that chromosome 15q25.3-26.2 may share a significant association with recurrent early-onset major depression, and this chromosome sequence mapping shows that this region may be significantly associated with SV2B (Verma et al., 2008; Nowack et al., 2010). In addition, previous research has indicated that pituitary miRNAs regulate growth hormone synthesis by targeting different GHRHR SVs (i.e., GHRHR, GHRHR SV1, and SV2) (Zhang et al., 2017; Cheng et al., 2020). Moreover, a recent study concluded that mutations in miR-96 might potentially influence the expression of SV2 (Schluter et al., 2018). Thus, we conclude that miR-96 could target and negatively regulate the expression of SV2C.

Furthermore, miR-96 inhibited SV2C expression to promote inflammation and oxidative stress in the brain, characterized by elevated levels of MDA, TNF-α, and IL-1β levels along with reduced SOD, GSH, 5-HT, and DA levels in LPS-treated mice. TNF-α has been well documented to play a chief role in the inflammatory cascade and body weight homeostasis (Muntyanu et al., 2020). TNF-α also plays an integral role in the pathophysiology of depressive disorders and the mechanism of antidepressant treatment (Ma et al., 2016). IL-1β has been highlighted as a crucial cytokine in the regulation of immune responses to infectious challenges and sterile insults (Wenjing et al., 2020). Previous research has emphasized MDA as an indicator of lipid peroxidation while suggesting that SOD is an enzyme that can eliminate harmful substances produced in the process of oxidative metabolism (Wang et al., 2019). GSH is the most abundant non-protein biothiol and is a central antioxidant to defend against the effects of toxins and free radicals in aging (Mao et al., 2019). 5-HT is a neurotransmitter derived from tryptophan that is synthesized both centrally and systemically (Sahu et al., 2018). 5-HT has been reported to modulate memory formation (Meneses, 2013), whereas the inhibition of DA reuptake attenuates depression-like behavior induced by traumatic brain injury in rats (Tan et al., 2015) and in other depression models. In light of this extended model, we now highlight the potential of miR-96 as a novel molecular marker of oxidative stress and immunoglobulin function (Ahmed et al., 2017).

## Conclusion

Taken together, the key findings of the current study provide evidence demonstrating that miR-96 inhibits the expression of SV2C to enhance the development of depression-like behavior and memory deficits. Based on the data obtained, miR-96 may prove to be a prognosis marker for depression-like behavior and memory deficits. Although our experiments successfully validate the effects of miR-96, there were some limitations experienced during our study. SV2A and SV2B paralogs of SV2 were mentioned during the study; however, we primarily focused on SV2C. At present, no notable literature exists in regard to the interaction between miR-96 and SV2A/SV2B, which should be considered in subsequent research. A further mechanistic investigation of this pathway should be performed with a diverse study population and the relevance of the miR-96 axis as a potential target for clinical depression.

## Data Availability Statement

The original contributions presented in the study are included in the article/Supplementary Material, further inquiries can be directed to the corresponding author.

## Ethics Statement

The animal study was reviewed and approved by the Animal Care and Use Committee of Ordos Fourth People’s Hospital.

## Author Contributions

LS, DB, ML, E, LZ, FW, and SJ designed the study. LS, DB, and ML collated the data, carried out data analyses, and produced the initial draft of the manuscript. E, LZ, FW, and SJ contributed to drafting the manuscript. All authors have read and approved the final submitted article.

## Conflict of Interest

The authors declare that the research was conducted in the absence of any commercial or financial relationships that could be construed as a potential conflict of interest.

## Footnotes

1 http://www.targetscan.org/vert_71/

2 http://www.mirdb.org/

3 http://www.rna-society.org/raid2/index.html

4 http://starbase.sysu.edu.cn/

5 http://diana.imis.athena-innovation.gr/DianaTools/

6 https://www.ncbi.nlm.nih.gov/gds

7 https://bioconductor.org/packages/limma/

8 https://mirmap.ezlab.org

9 https://cm.jefferson.edu/rna22/Precomputed/
