# Supplementary figures and images for: miR-96 Inhibits SV2C to Promote Depression-Like Behavior and Memory Disorders in Mice
Source: Front Behav Neurosci. 2021 Mar 19;14:575345. doi: 10.3389/fnbeh.2020.575345 (PMC8017146; doi:10.3389/fnbeh.2020.575345)

## SUPPLEMENTARY MATERIAL

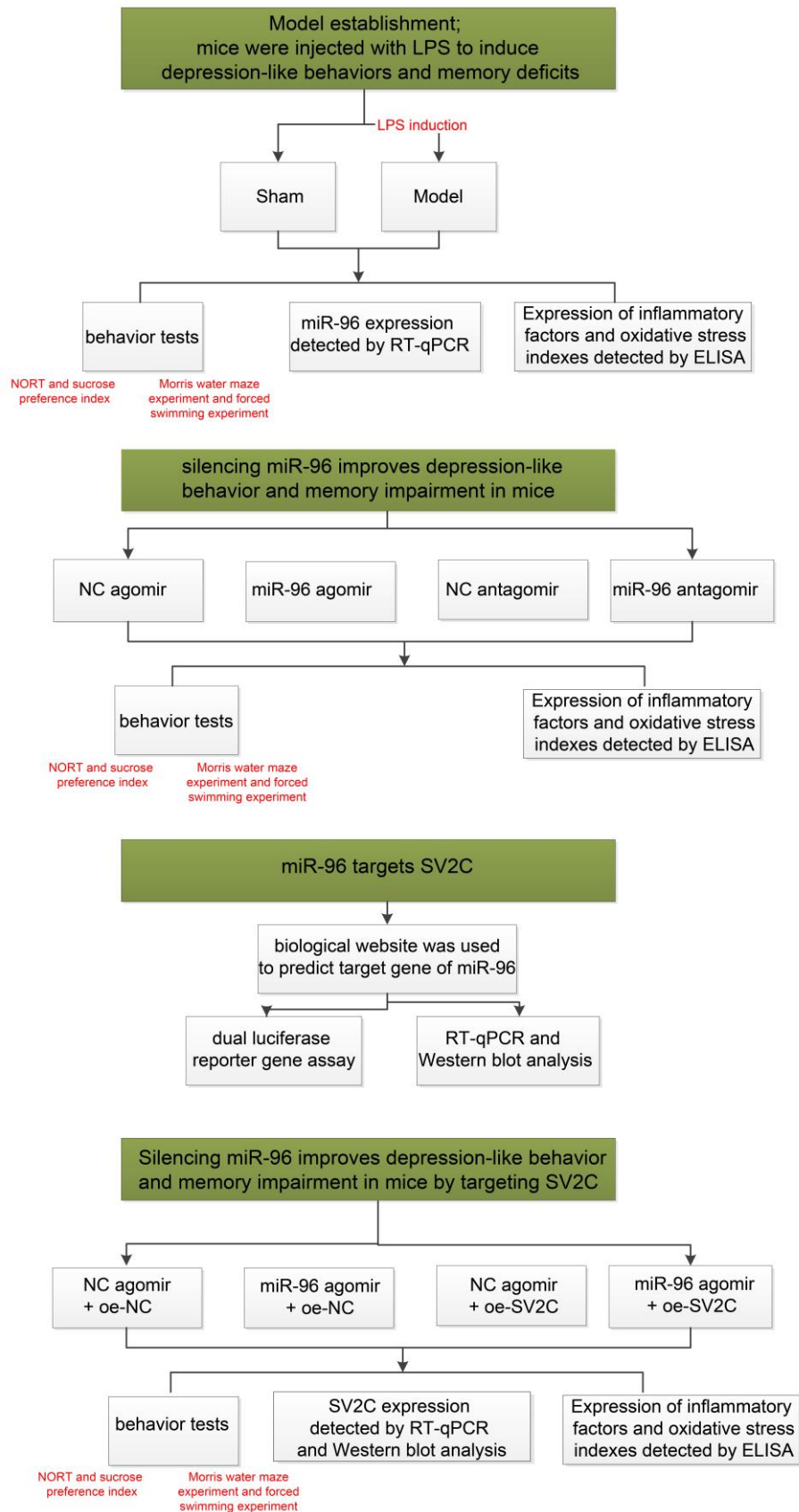

**SUPPLEMENTARY FIGURE 1** Study design schematics.

Supplement: Supplementary file 1 [file Image_1.pdf]
